# A Cell Model for Conditional Profiling of Androgen-Receptor-Interacting Proteins

**DOI:** 10.1155/2012/381824

**Published:** 2012-02-27

**Authors:** K. A. Mooslehner, J. D. Davies, I. A. Hughes

**Affiliations:** Department of Paediatrics, Addenbrooke's Hospital, University of Cambridge, Level 8, Box 116, Hills Road, Cambridge CB2 0QQ, UK

## Abstract

Partial androgen insensitivity syndrome (PAIS) is associated with impaired male genital development and can be transmitted through mutations in the androgen receptor (AR). The aim of this study is to develop a cell model suitable for studying the impact AR mutations might have on AR interacting proteins. For this purpose, male genital development relevant mouse cell lines were genetically modified to express a tagged version of wild-type AR, allowing copurification of multiprotein complexes under native conditions followed by mass spectrometry. We report 57 known wild-type AR-interacting proteins identified in cells grown under proliferating and 65 under nonproliferating conditions. Of those, 47 were common to both samples suggesting different AR protein complex components in proliferating and proliferation-inhibited cells from the mouse proximal caput epididymus. These preliminary results now allow future studies to focus on replacing wild-type AR with mutant AR to uncover differences in protein interactions caused by AR mutations involved in PAIS.

## 1. Background

Androgen insensitivity gives rise to a wide spectrum of disorders in man, the most severe being complete sex reversal, to milder forms of PAIS associated with ambiguous or underdeveloped genitalia, or even milder forms causing “only” male infertility in otherwise healthy males. Often mutations in the androgen receptor (AR) are involved which interfere with ligand binding, DNA binding, or increase or decrease intramolecular interactions between AR domains [1]. Where no mutations are identified in the AR [2] mutations in AR coregulators may be implicated in failure to activate or repress androgen-regulated target genes. Although there are a number of mouse models available to study impaired AR function *in vivo *[3], the signalling networks are too complex to dissect without using simpler cell models. The aim of this study was to develop a cell model for the study of AR signalling in the urogenital tract. In turn this may identify disrupted signalling resulting from AR mutations associated with PAIS. Male genital development relevant murine cell lines PC1 (proximal caput epithelial cells from mouse epididymus) [4] and MFVD (mesenchymal fetal vas deferens cells) [5] were genetically modified to express a tagged wild-type AR to test the system. The modifications allow purification of multiprotein complexes associated with AR under native conditions and analysis of the copurified protein complexes by mass spectrometry. The data was analysed using readily available bioinformatics software: the pathway mining tool of “DAVID” bioinformatics resources [6, 7] and the gene group functional profiling tool of g : profiler [8]. By focussing solely on known AR coregulators, we were able (as a proof of principle) to uncover differences in the proteome of proliferating and nonproliferating epithelial PC1 cells.

## 2. Methods

### 2.1. N-Terminal Tandem Affinity Purification Tag (N-TAP)

**Figure 1:**
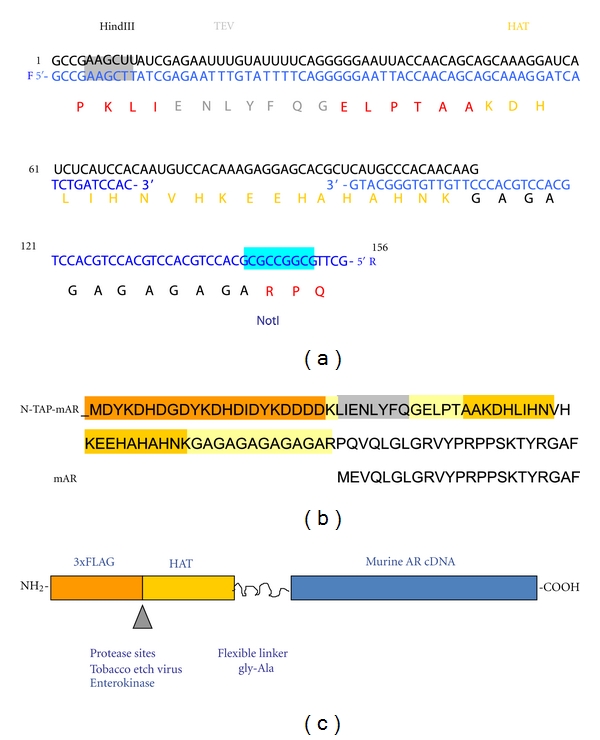
Creation of the mouse N-TAP-mAR. (a) Amplification of the TEV-HAT-glycine alanine repeat sequence with primers F and R using the C-TAP from Fernández et al., 2009 [9] as template. (b) Aminoacid sequence of the complete NH2-terminus of the androgen receptor. The N-TAP increases the size of the mAR by 70 amino acids and the molecular weight by 7.8 kDa (http://web.expasy.org/compute_pi/). (c) Schematic showing the tagged androgen receptor (N-TAP-mAR) cDNA clone. The N-terminal TAP tag was selected on the basis of small size. It contains three FLAG epitopes (3xFLAG) and 6xHistidine residues (HAT) located in an alpha helix.

The N-TAP was designed by modifying the C-terminal tandem affinity tag (C-TAP) from Fernández et al., 2009 [9]. The HAT tag was amplified, using the C-terminal tag [9] as a template and PCR primers (Figure 1(a)) designed to add the TEV protease cleavage site with the forward primer and the glycine-alanine repeat and an additional NotI-cloning site with the reverse primer. The HindIII-NotI fragment was then inserted into the polylinker region of p3XFLAG-CMV-10 (Sigma) in frame with 3XFLAG (Figure 1(b)). The mouse androgen receptor cDNA clone (gift from Professor Jan Trapman, Department of Pathology, Erasmus MC/JNI Rotterdam) was modified by replacing the start methionine ATG with an NotI site-by-site directed mutagenesis (Stratagene) and introducing the 2.787 kb NotI-BHI full length mouse cDNA into the N-TAP-CMV-10 vector (Figure 1(c)). The N-TAP-mAR fusion construct was confirmed by sequencing.

### 2.2. Transient Transfection and Luciferase Assay

In total 10^5^ COS-1 or Hela cells/well were seeded into 12-well tissue culture plates in DMEM, containing 10% charcoal-stripped serum. Cells were transiently transfected using Fugene (Roche) or Lipofectamine 2000 (Invitrogen) with 25 ng AR or N-TAP-mAR, 500 ng of pGRE-luciferase and 25 ng pTK-RL according to manufacturer's instructions. 12–16 hours after transfection, the medium was replaced with DMEM, containing 10% charcoal-stripped serum + or −10 nM dihydrotestosterone (DHT; Sigma). 24 h later, cells were harvested and lysed in 25 mM glycine (pH 7.8), 15 mM MgSO_4_, 4 mM EGTA, 1% triton × 100 and 1 mM dithiothreitol. Luciferase assays were performed with reagents from nanolight technology and the ratio of luciferin : renillaluciferase activity was measured using a Turner TD-20/20 luminometer. Standard error bars relate to three independent transfection experiments.

### 2.3. Control Cell Lines

Androgen responsive cell lines PC1 and MFVD from the mouse urogenital tract served as control cell lines in their nonmodified state. The PC 1 cell line was a gift from Araki et al. [4] and the MFVD cell line a gift from Umar et al. [5]. The PC1 cell line is an epididymal cell line immortalized with SV 40 large T-antigen and has been characterized in great detail regarding morphology [4], epithelial and epididymus specific gene expression [4], and androgen responsiveness [10]. Also the MFVD cell line is immortalized by expression of a temperature sensitive SV 40 large T-antigen but of mesenchymal origin. MFVD cells were derived from fetal (18 *d.p.f.*) mouse vas deferens and show features of Wolffian duct mesenchymal cells and androgen responsiveness [5]. Both cell lines were continuously cultured under conditions given elsewhere [4, 5] in the presence of 5 nM mibolerone, a synthetic androgen.

### 2.4. Establishment of Stable Cell Lines

The control cell lines PC1 and MFVD were transfected with the ScaI (unique site in the bacterial ampicillin resistance) linearised N-TAP-mAR vector using Lipofectamine 2000 (Invitrogen). After 48 hours, transfected cells were replated in dilutions from 10^5^–10^3^ cells/14 cm diameter dish and G418 resistant clones were selected with 750 *μ*g/mL G418 (PC1) and 250 ug/mL G418 (MFVD). Single colonies coming up were picked with cloning rings, grown up and tested for N-TAP-mAR expression by Western blot analysis and immunocytochemistry using the FLAG M2 antibody (Sigma) and the AR-N20 antibody (Santa Cruz).

### 2.5. Growth of Proliferating and Nonproliferating PC1 and P17 Cells

PC1 cells, derived from the mouse proximal caput epididymus and immortalized by expression of a temperature sensitive SV 40 large T-antigen, were continuously cultured at 33°C, the permissive temperature of large T in the presence of 5 nM mibolerone. At physiological temperature (37°C), cell growth is partly inhibited and T-antigen-expressing cells can survive [11]. Large T is however degraded after prolonged exposure of the cells to the nonpermissive temperature (39°C), then significant cell death occurs and the cells do not recover [11]. To prevent cell death but still have an inhibitory effect on proliferation early passage PC1 and P17 cells were cultured to near confluence at 33°C and kept then for 1 week at 37°C in growth medium containing 5 nM mibolerone before preparing cytoplasmic and nuclear extracts of the still healthy looking cells. For extracts made from proliferating PC1 and P17 cells, the cells were grown at 33°C in growth medium containing 5 nM mibolerone. Similar amounts of cell pellets from proliferating and nonproliferating cells were processed for protein extraction.

### 2.6. Immunoblotting

Cells from subconfluent cultures were washed, trypsinized, and pelleted by centrifugation and washed 3x in cold PBS. For whole cell lysates, cells were lysed in SDS loading buffer (0.03125 M Tris pH 6.8, 5% glycerol, 0.001% bromphenol blue 3, 1% SDS, 2.5%  *β*-mercaptoethanol). Samples were subjected to sodium dodecyl sulphate-polyacrylamide gel electrophoresis (SDS-PAGE), transferred to polyvinylidene difluoride membrane, blocked and probed in phosphate-buffered saline (150 mM NaCl, 3 mM KCl, 10 mM phosphate salts (mono and dibasic) (pH 7.3), 0.05% (vol/vol) Tween 20) containing 10% nonfat dry milk. Primary antibodies were used at recommended dilutions and horseradish peroxidase-conjugated secondary antibodies (Dako) and ECL Plus Blotting Detection Reagents (Amersham) or SuperSignal West Femto (Thermo Scientific) were used as described in the manufacturer's instructions. SRC-1 (128E7) and CTNNB1 (9587) antibodies were purchased from NEB, FLAG M2 (F3165) from Sigma, and N20-AR (Sc-816), NR3C1 (Sc-8992), SMARCC1 (Sc-9748), ACTB (Sc-81178) from Santa Cruz.

### 2.7. Cytoplasmic and Nuclear Protein Extraction and Purification of N-TAP-mAR

The protein extraction protocol is a modified version of a chromatin extraction protocol for co-purification of histones by Saade et al., 2009 [12]. Cells were grown on 14 cm diameter dishes to confluence (10 plates PC1 or 20 plates P17 gave about 700 *μ*L cell volume), washed 3x with prewarmed PBS, trypsinised with 1 mL trypsin-EDTA (Sigma)/plate 5 minutes 37°C, inactivated with medium, pooled in 50 mL Falcon, spun1200 rpm 5 mins RT, and washed 3x with cold (4°C) PBS. Cells were transferred to a 2 mL microfuge tube and pelleted by centrifugation at 6500 rpm 2-3 minutes at 4°C. Pellets (2 × 200 *μ*L) were resuspended in 2 × 1.8 mL hypotonic buffer (10 mM Tris pH 7.5, 10 mM KCl, 1.5 mM MgCl, 0.1% Triton, 4.5 mM *β*-mercaptoethanol, protease inhibitor cocktail (Roche), 5 nM mibolerone, 1 mM Pefabloc (Roche), and Phosstop (Roche)) by pipetting up and down and vortexing vigorously on highest setting for 15 seconds followed by 45 minute incubation on ice vortexing every 10 minutes. Nuclei were spun down at 4°C 13.000 RPM for 5 minutes, the supernatant, called here cytosol fraction, transferred to a clean tube and NaCl was added to 15 mM. This cytosol fraction was added to FLAG-M2 coupled magnetic beads (ca. 5 mg) after keeping an aliquot as cytosol input fraction for PAGE. Coupling of the magnetic beads (Dynabeads M-270 Epoxy from Invitrogen) to the FLAG M2 antibody (Sigma F3165) was performed according to manufacturer's instructions. Cytosol extracts on beads rotated for 1 hour minimum at 4°C. Nuclei were taken up in sucrose buffer (0.34 M sucrose, 10 mM Tris pH8, 3 mM MgCl_2_, 1 mM CaCl_2_, 150 mM NaCl, 1 mM DTT, protease inhibitor cocktail (Roche), 5 nM mibolerone, 1 mM Pefabloc (Roche), Phosstop (Roche)) 150 *μ*L/100 *μ*L cell pellet and resuspended. 0.3 units micrococcal nuclease from Sigma (0.1 U/*μ*L in 10 mM Tris/0.1 mM CaCl_2_ pH8) for 100 *μ*L nuclear extract were added and incubated for 30 min at 37°C. Extracts were then diluted with 1 volume of sucrose buffer and sonicated 6 × 10 seconds on ice with 10 sec bursts and 10 sec cool downs alternating. Extracts were finally loaded onto FLAG M2-coupled magnetic beads and rotated for at least 1 hour at 4°C in cold room. Supernatants were kept as flow through and aliquots before loading on to the beads as nuclear input. After the incubation period, beads loaded with cytosol preparations were washed 3x with hypotonic wash buffer (10 mM Tris pH 7.5, 10 mM KCl, 1.5 mM MgCl, 0.1% Triton, 4.5 mM *β*-mercaptoethanol, protease inhibitor cocktail (Roche), 5 nM mibolerone, 1 mM Pefabloc (Roche) and Phosstop (Roche), 0.5% NP40, 0.05% sodium deoxycholate, 0.005% SDS, 15 mM NaCl) or sucrose buffer (nuclear preps) followed by 3 washes with TEV buffer (0.05 M Tris pH 8, 1 mM DTT, 0.5 mM EDTA) supplemented with Protease Inhibitor Cocktail (Roche), mibolerone (5 nM), and phosphatase inhibitors (Phosstop and Pefabloc (Roche), and NaCl to15 mM NaCl in cytosolic and 150 mM NaCl in nuclear TEV buffer. The beads were finally taken up in 100 uL TEV buffer and bound protein complexes were eluted with 10 units of TEV protease (GST-tag) (TEVP US Biological) at RT for 1 hour. Protein concentration in the eluted fraction was determined by Bradford assay. The beads were kept in PBS for recycling.

### 2.8. Mass Spectrometry

Colloidal coomassie stained protein bands (Colloidal Blue Staining Kit, Invitrogen) were excised out of a 5% PAGE after separation in 7 slices covering a size spectrum of 48 kDa to the top of the gel. Gel slices were briefly washed in MilliQ water and kept in water at −80°C until submitted to “Cambridge Proteomics Services” where all LC-MS/MS experiments were performed using an Eksigent NanoLC-1D Plus (Eksigent Technologies, Dublin, CA) HPLC system and an LTQ Orbitrap Velos mass spectrometer (ThermoFisher, Waltham, MA). Separation of peptides was performed by reverse-phase chromatography used at a flow rate of 300 nL/min and an LC-Packings (Dionex, Sunnyvale, CA) PepMap 100 column (C18, 75 *μ*M i.d. × 150 mm, 3 *μ*M particle size). Peptides were loaded onto a precolumn (Dionex Acclaim PepMap 100 C18, 5 *μ*M particle size, 100A, 300 *μ*M i.d. × 5 mm) from the autosampler with 0.1% formic acid for 5 minutes at a flow rate of 10 *μ*L/min. After this period, the valve was switched to allow elution of peptides from the precolumn onto the analytical column. Solvent A was water + 0.1% formic acid and solvent B was acetonitrile + 0.1% formic acid. The gradient employed was 5–50% B in 45 minutes. The LC eluant was sprayed into the mass spectrometer by means of a new objective nanospray source. All *m/z* values of eluting ions were measured in an Orbitrap Velos mass analyzer, set at a resolution of 30000. Data dependent scans (Top 20) were employed to automatically isolate and generate fragment ions by collision-induced dissociation in the linear ion trap, resulting in the generation of MS/MS spectra. Ions with charge states of 2+ and above were selected for fragmentation.

### 2.9. Data Processing and Database Searching

After run, the data were processed using Protein Discoverer (version 1.2., ThermoFisher). Briefly, all MS/MS data were converted to mgf (text) files. These files were then submitted to the Mascot search algorithm (Matrix Science, London UK) and searched against Uniprot Mouse database, using a fixed modification of carbamidomethyl and variable modifications of oxidation (M).

## 3. Results

### 3.1. Isolation of N-TAP-mAR Complex in MFVD and PC1 Cell Lines

**Figure 2:**
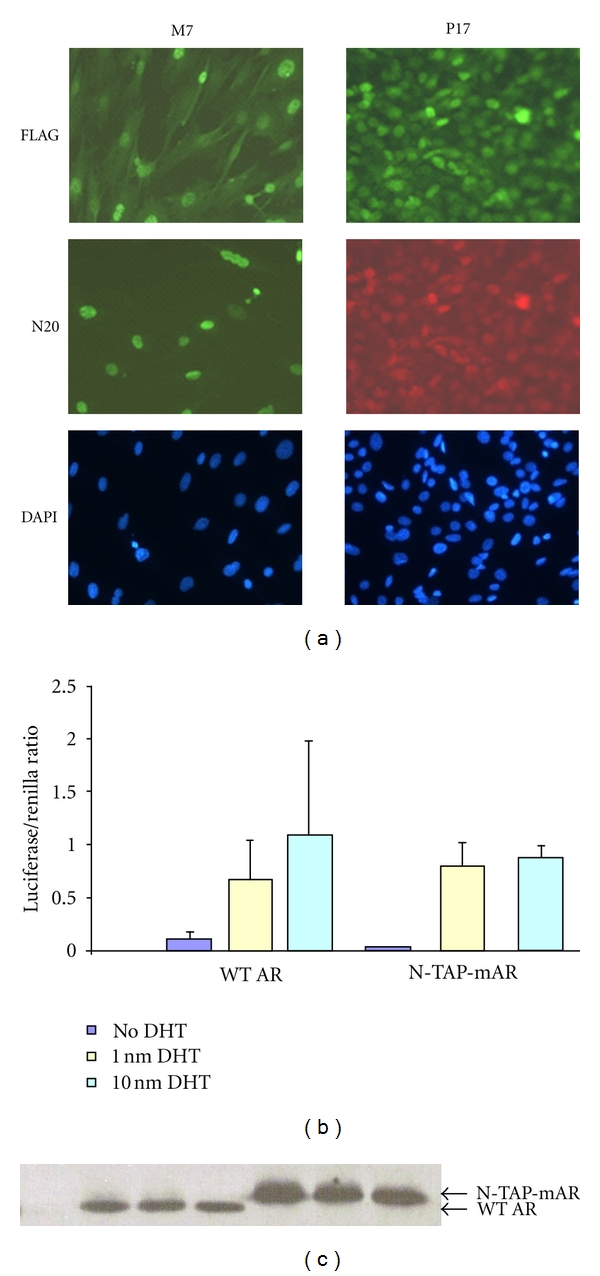
Nuclear localization and transactivation ability of N-TAP-mAR. (a) Immunostaining of a stable mesenchymal cell line M7 and a stable epithelial cell line P17 expressing N-TAP-mAR. The FLAG antibody detects tagged androgen receptor only. N20 antibody recognises both endogenous AR and N-TAP-mAR. Nuclei staining with DAPI indicates the percentage of cells expressing NTAP-mAR. (b) The ability of the modified N-TAP-mAR and WT-AR to activate a glucocorticoid response element (GRE) was assayed in COS-1 cells in a transient transfection luciferase assay. GRE promoter activation, shown here on the *y*-axis as luciferase/renilla ratio, was similar for the tagged and nontagged androgen receptor. (c) Tagged androgen receptor expression (N-TAP-mAR) was elevated in the Western blot done with protein extracts from the transactivation samples relative to WT.

Stable MFVD and PC1 clones expressing N-TAP-mAR were developed as described in Section 2. Total protein was extracted from several clones and analyzed for N-TAP-mAR expression by Western blot using FLAG and AR-specific antibodies (data not shown). A second criteria was the nuclear localization of N-TAP-mAR, which was examined in several clones by immunofluorescence using FLAG and AR-specific antibodies. One MFVD cell line (M7) and one PC1 cell line (P17) were selected on the basis that they expressed similar levels of stably integrated N-TAP-mAR and endogenous wild-type AR (WT AR). Both were predominantly located in the nucleus when grown in medium supplemented with 5 nM mibolerone (Figure 2(a)). The transactivation properties of N-TAP-mAR were tested in COS cells by cotransfection of a GRE reporter construct, with expression levels confirmed by Western blot (Figures 2(b) and 2(c)). Levels of expression are stable, allowing considerable grow up of cells. 

**Figure 3:**
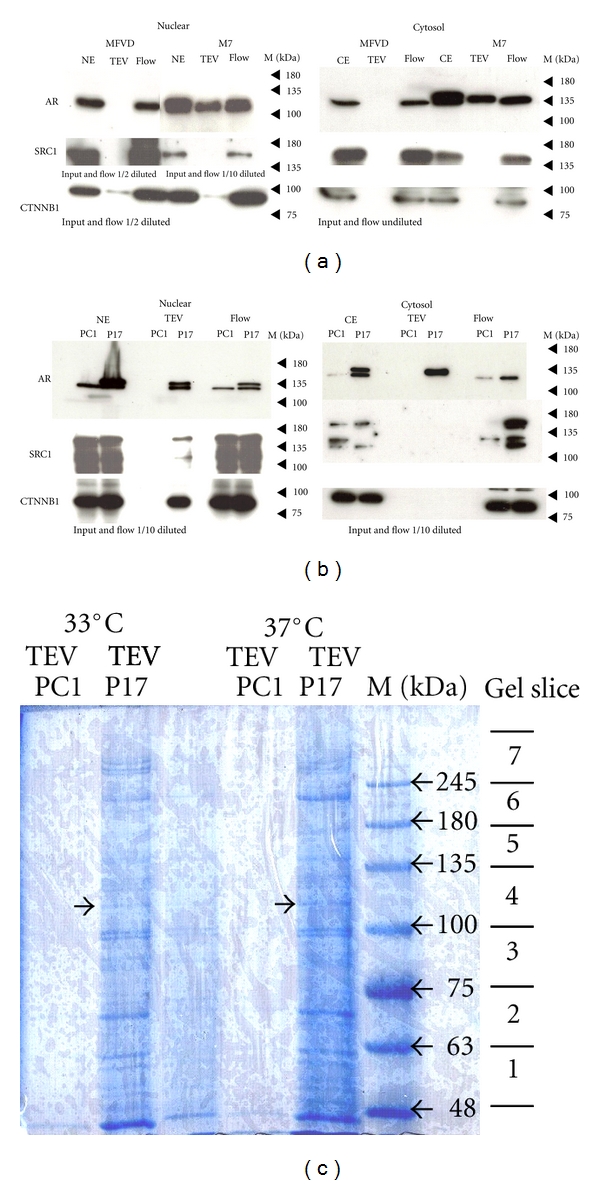
FLAG purifications. (a) Western blot analysis of FLAG-purified M7 nuclear and cytosol extracts using TAP negative MFVD cells as control. Compared are SRC1, beta catenin (CTNNB1), and AR expression in extracts before (NE/CE) and after (FLOW) purification and in the TEV-eluted fractions. AR (WT and N-TAP-mAR comigrate) is recovered in TEV eluate from the M7 but not from the MFVD control purifications. (b) Western blot analysis of FLAG-purified P17 nuclear and cytosol extracts using TAP negative PC1 cells as control. Compared are SRC1, beta catenin, and AR expression in extracts before (NE/CE) and after (FLOW) purification and in the TEV-eluted fractions. AR (WT and N-TAP-mAR are here distinguishable) is recovered in TEV eluate from the P17 but not from the PC1 control purifications. (c) Coomassie stained 5% PAGE loaded with TEV-eluted fractions from the control cell line PC1 and the NTAP-mAR expressing cell line P17 grown at 33°C and 37°C after FLAG purification. This gel was prepared for cutting out gel slices (1–7), which were then submitted for mass spectrometry. The arrows indicate the N-TAP-mAR migration. M is the size estimate by a prestained protein ladder in kilo Daltons (kDa).

Different protein purification protocols for producing nuclear and cytosol cell extracts were tested. Commercially available purification kits and a modified version of a chromatin purification protocol (FLAG-antibody capture) were compared and the latter was chosen. FLAG-antibody capture gave a good recovery of N-TAP-mAR when preparing extracts from M7 (Figure 3(a)) or P17 cells (Figure 3(b)). The known coregulators SRC-1 and CTNNB1 were copurified in P17 extracts but not in M7 extracts. Attempts to optimize His-tag purification conditions were unsuccessful; Nickel affinity purification of the PC1 (N-TAP-mAR negative control) chromatin extracts gave high background with unspecific protein binding to the Nickel resin. The cell lines proliferate at 33°C with growth driven by temperature sensitive SV40 T-antigen; and proliferation stops at 37°C.  Figure 3(c) illustrates a preparative gel of TEV protease-eluted proteins purified from the nuclei of P17 cells cultured at 33°C and 37°C. The gel slices were selected for LC- MS/MS, and data processed using the Mascot Search engine at the Cambridge Centre for Proteomics.

The results from the LC-MS/MS search are portrayed as peptide matches and grouped as protein hits using a simple parsimony algorithm (http://www.matrixscience.com/). Only those ions scores that exceed a significance threshold of 0.05 (1 in a 20 chance of being a false positive) contributed to the score. This would translate into 1500 peptides falling within the mass tolerance window to have a score of ≥45. Known AR-interacting proteins (http://androgendb.mcgill.ca) were identified among the protein hits and are listed in Table 1 along with their respective peptide scores. In this first mass spec analysis, we identified 1196 AR associated proteins in FLAG purified extract from proliferating cells and 1456 from nonproliferating cells. Of those 882 were common between those two groups, 314 were only picked up in FLAG purifications from proliferating cells and 574 were only picked up in FLAG purifications from nonproliferating cells. Functional profiling was carried out using web tools “DAVID” [6, 7] and “g : profiler” [8], providing functional enrichments in the form of pathways, biological processes, molecular functions, metabolic functions, cellular localization, protein-protein interactions, and shared transcription factor binding sites. Only pathways and biological processes which received the highest scores are utilized.

### 3.2. Analysis of Gene Lists with DAVID Bioinformatics Resources [6, 7]

**37°C**

The gene list of known AR-interacting proteins identified in the nuclear FLAG purifications of the 37°C samples (37 only + common, Table 1) was accepted as 65 DAVID ID's using DAVID bioinformatics resources [6, 7]. Gene ontology tool “GOTERM_BP_FAT” gives the highest score to the biological process (BP) “regulation of transcription” with 40 contributing genes (Table 2). Another high scoring biological process “chordate embryonic development” listed 11 genes: NCOR2, SMARCA4, AR, PSMC3, PRKDC, KDM1A, TRP53, and EP300 common to both temperatures and MED1, NF1, and SP1 specific to the 37°C samples (Table 4). When analyzing the gene list for pathways, 10 genes are components of the Kegg Pathway: pathways in cancer: EP300, AR, CTNNB1, DAPK3, HSP90, PIAS1, RB1, STAT3, HDAC1, and TRP53. Of these 10 genes only STAT3, RB1, and DAPK3 have inhibitory function in this pathway. 6 of those 10 genes (EP300, AR, CTNNB1, Hsp90, RB1, and TRP53) have been associated with prostate cancer (Table 3).

The BIOCARTA Chart revealed overrepresented pathways: “telomeres, telomerase, cellular aging, and immortality”, with 5 genes (XRCC5, XRCC6, HSP90, RB1, and TRP53) involved. Another overrepresented pathway “chromatin remodeling by hSWI/SNF ATP-dependent complexes” involves genes SMARCA4, ACTB, NR3C1, NF1, ARID1A. A third overrepresented BIOCARTA pathway is “control of gene expression by vitamin D receptor” with EP300, SMARCA4, MED1, NCOA2, and ARID1A involved (Table 3). All 3 pathways do not include the androgen receptor.

**33°C**

The Gene list of known AR-interacting proteins identified in the nuclear FLAG purifications of the 33°C samples (33 only + common, Table 1) was accepted as 56 DAVID ID's using DAVID bioinformatics resources [6, 7]. ARID1B was not detected as DAVID ID and therefore not included in the analysis. As with the samples from 37°C, the gene ontology tool “GOTERM_BP_FAT” gives the highest score to the biological process (BP) “regulation of transcription” with 34 contributing genes (Table 2). Looking at overrepresented pathways only 7 AR-interacting proteins are components of the Kegg pathway “pathways in cancer”: EP300, AR, CTNNB1, HSP90, STAT3, HDAC1, and TRP53. Proteins RB1, DAPK3, and PIAS1 are not present in the 33°C samples (Table 3). Following on from that, only 5 of those genes have been associated with prostate cancer (EP300, AR, CTNNB1, HSP90, and TRP53) and the inhibitory function of RB1 is missing (Table 3).

Again the BIOCARTA Chart brings up as overrepresented pathways: “telomeres, telomerase, cellular aging, and immortality”, but here with only 4 genes being involved (XRCC5, XRCC6, HSP90, and TRP53) and RB1 missing. “Chromatin remodeling by hSWI/SNF ATP-dependent complexes” has NR3C1 and NF1 missing and gained 3 new components with ARID1B, SMARCC1, and SMARCD1. SMARCA4, ACTB, and ARID1A are present. The pathway “control of gene expression by vitamin D receptor” has now 5 components present EP300, SMARCA4, but not MED1 and NCOA2 anymore. ARID1A is still present and the 2 new components SMARCC1 and SMARCD1.

### 3.3. Analysis of Gene Lists with g : Profiler [8]

Identical gene lists submitted to DAVID were also submitted to the gene ontology online tool g : Profiler for functional characterization. “Regulation of transcription” was not a high scoring biological process” on this occasion, whereas “gene expression” scored high at both temperatures, with many genes overlapping in both categories (Table 2). Also here the Kegg pathway components for prostate cancer are revealed for both temperatures, but only 4 (HSP90, AR, CTNNB1, and TRP53) as shown in Table 3.

**37°C**

Kegg pathway: “pathways in cancer” is only detected in the 37°C samples, with 9 components represented (EP300, STAT3, HDAC1, HSP90, AR, CTNNB1, TRP53, DAPK3, and PIAS1). The gene ontology subgroups for “biological processes” (BPs), “anatomical structure morphogenesis”, and “embryo development” are overrepresented in the 37°C samples and overlapping components are involved (Table 4). “Embryo development” components are NCOR2, AR, CTNNB1, KDM1A, PRKDC, SP1, MED1, SMARCA4, HDAC1, TRP53, TGFB1l, PSMC3, and NF1. “Anatomical structure morphogenesis” components are NCOR2, AR, CTNNB1, ACTB, PRKDC, SP1, MED1, SMARCA4, HDAC1, TRP53, TGFB1l, GSN, NF1, GATA3, STAT3, NR3C1, and STAT3. Comparison of these 2 groups suggests a more specific function in morphogenesis during embryo development for NR3C1, GATA3, and STAT3 (strong evidence) GSN and ACTB (weak evidence).

**33°C**

Among cellular component profiling, the nBAF complex is solely detected in the 33°C samples with SMRCC1, SMARCD1, SMRCA4, and ARID1A (not listed in tables). These proteins are also components of the SWI/SNF chromatin remodelling complex.

### 3.4. Western Blot Analysis of Cofactors Identified Only in Proliferating or Only in Nonproliferating PC1 Cells

**Figure 4:**
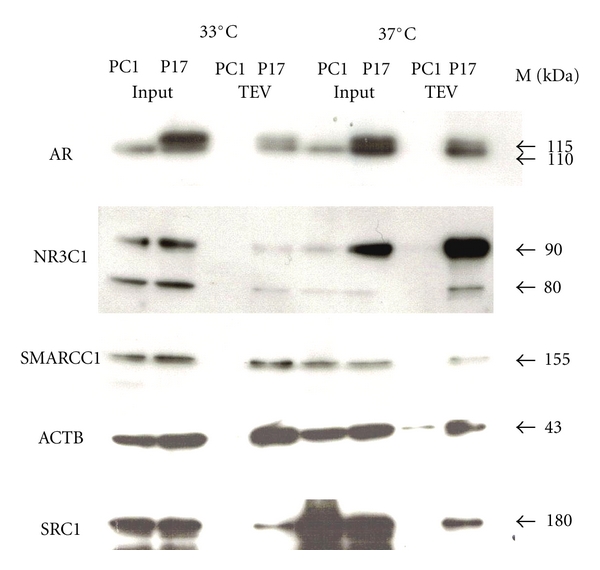
AR interacting proteins SMARCC1 and NR3C1 are differentially expressed in proliferating versus nonproliferating P17 cells. Western blot analysis of known AR coregulators identified by mass spectrometry only in AR FLAG copurifications from proliferating P17 cells (SMARCC1) and of a known coregulator identified by mass spectrometry only in AR FLAG copurifications from nonproliferating P17 cells (NR3C1). SRC1 was not identified at all, neither in purifications from proliferating nor nonproliferating P17 cells. AR and ACTB were identified in both FLAG copurifications from P17 cells by mass spectrometry. Compared to the other identified known AR interacting proteins NR3C1, SMARCC1, and ACTB, a smaller portion of the total SRC1 present in the nuclear extract (P17 Input) interacts with AR (P17 TEV). M is the size estimate by a prestained protein ladder in kilo Daltons (kDa).

The observation that some AR-interacting proteins were only detectable in proliferating cells and others only in nonproliferating cells (Table 1) could simply mean that the quality of the cell extracts varies and much less protein is present in one of the extracts. Another reason might be that expression levels of those proteins alter during cell proliferation, and less or more protein is available for interaction with AR. A third option is that the binding affinity of AR to the interacting proteins changes with the proliferation status of the cells. To address these questions, we carried out Western blots, probing those AR-interacting proteins which were differentially expressed (Figure 4).

No major differences in expression were observed when extracts were probed with AR-N20 and ACTB control antibodies, but differences were found elsewhere. NR3C1 is expressed at much higher levels in nonproliferating versus proliferating P17 cells, and expression levels of SMARCC1 were slightly lower in nonproliferating (37°C) PC1/P17 when compared to proliferating cells (33°C). The difference in the amounts of AR copurified SMARCC1 in proliferating versus nonproliferating is not as striking as it is for NR3C1, and the amounts at “low expression” temperatures are probably too small to be detected by mass spectroscopy. The proliferation status of P17 cells seems to affect SMARCC1 and NR3C1 expression levels per se as SMARCC1 expression is up and NR3C1 expression is down in proliferating cells. However, the loss of NR3C1 expression in proliferating cells is unlikely to account for all the loss in AR binding observed here. Similar loss of SMARCC1 expression in nonproliferating cells does not account for all the loss in AR binding detected. Therefore, expression levels of coregulators and binding affinity to AR may contribute to the differences observed in the 33°C and 37°C coregulator profiles. The N-TAP-mAR itself appears to increase GR expression levels by at least 3 fold under nonproliferating conditions comparing GR expression in PC1 and P17 input samples (Figure 4).

## 4. Discussion

We have developed the epithelial and mesenchymal mouse cell lines P17 and M7 for copurification of AR-associated protein complexes under native conditions. The two cell lineages are derived from the proximal caput epididymus (P17) and the mesenchyme of the fetal vas deferens (M7) of the mouse. The decision to carry out preparative scale N-TAP-mAR purification on PC1 cells rather than MFVD cells was based on the observation that CTNNB1 and SRC-1 could not be copurified from MFVD cells with our extraction protocol, whereas in P17 copurifications both coactivators were easily detected. Another advantage of choosing the PC1 cell line was the more abundant AR expression and the rapid growth compared to the mesenchymal cells. One confluent dish of PC1 gave about 5–10 times as much cell pellet than 1 confluent dish of MFVD.

To demonstrate proof of principle we used the newly developed purification protocol to detect differences in AR cofactor binding in cells grown under proliferating (33°C) and nonproliferating (37°C) conditions. As expected, many of the copurified proteins confirm the role of the AR in chromatin remodelling machinery, transcriptional complexes and associated with the cytoskeleton. The purification protocol we use is relatively crude and results in enrichment of 200–500 bp DNA fragments after a micrococcal nuclease digestion. The isolation protocol is nondenaturing and keeps the chromatin fraction as intact as possible. No size fractionation step is included, which could allow isolation of larger chromatin fractions, especially heterochromatic fractions which are not degraded by micrococcal nuclease. This fraction could contribute to a background of unspecific binding, which is difficult to control for as this might not occur in the PC1 control sample, where the “anchor” in form of the N-TAP-mAR is missing. On the other hand, the purification protocol is selecting for stable interactions, because no cross-linking step is included in our purification procedure.

We have not tested whether novel interacting proteins identified with this approach are indeed associated with AR or whether they are just contaminants. These potential interacting proteins could be novel AR-interacting proteins, but could also bind unspecific to the FLAG-M2-coupled magnetic beads. Unspecific binding to the FLAG antibody-coupled magnetic beads is estimated at 5% based on the recovery of protein from the FLAG purification of the PC1 control sample. This would mean that 1 out of 20 identified proteins is not part of the AR-associated complex. Unfortunately we were not able to reduce this background with an additional His purification step, because the FLAG purified and protease (TEV) eluted fraction was not able to specifically bind Nickel resin, probably caused by complex components covering up the His tag. In this study we concentrate only on a small proportion of all the potential AR-binding partners identified, representing the already known AR-interacting proteins. We show that our approach has the potential to differentiate between proteins which preferably form part of the AR complexes in proliferating or nonproliferating conditions, and those proteins where interaction is independent of the proliferation status of the cells.

### 4.1. Functional Enrichments among the Known AR Cofactors Identified with Two Different Bioinformatics Resources

**Figure 5:**
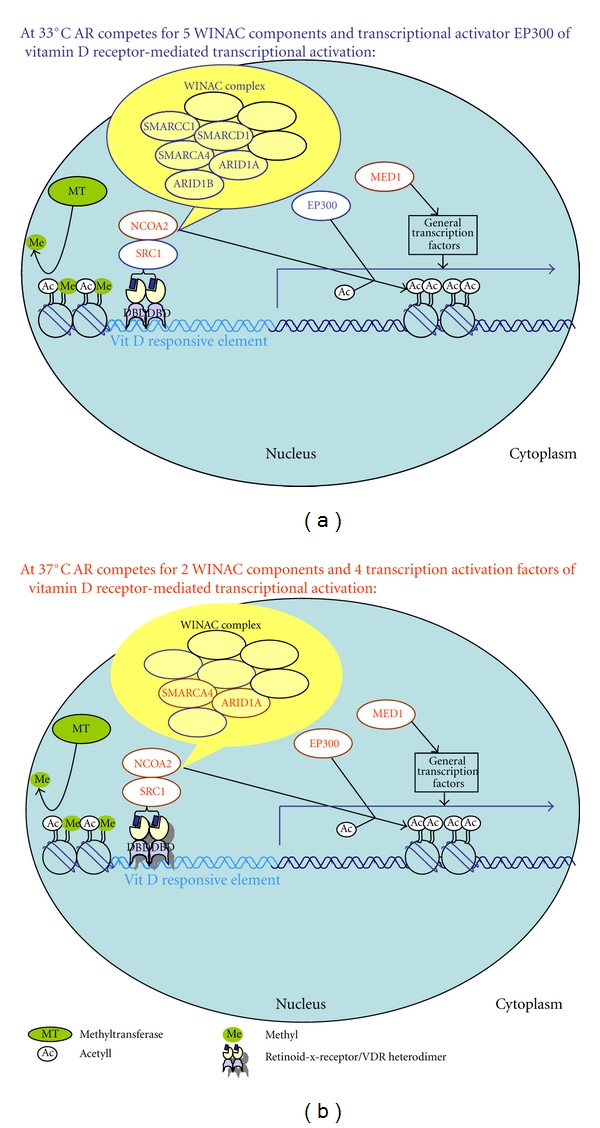
AR-interacting proteins are WINAC complex components and transcriptional activators of VDR-mediated gene expression. Presented is a simplified version of the BIOCARTA pathway “control of gene expression by Vitamin D receptor (VDR)” illustrating how AR and VDR may compete for shared coregulators. (a) In AR copurifications from proliferating P17 cells (33°C), the transcriptional activators EP300, SRC1*, and 5 proteins from the ATP, dependent chromatin remodelling complex WINAC: SMARCA4, ARID1A, ARID1B, SMARCC1, and SMARCD1 were identified. Under proliferating conditions, AR would therefore compete with VDR for 5 WINAC components and for 2 transcriptional activators. (b) In copurifications from nonproliferating P17 cells (37°C) only 2 known AR-interacting proteins identified were components from the WINAC complex: SMARCA4 and ARID1A. However, 4 proteins identified: EP300, NCOA2, MED1, and SRC1* act as transcriptional activators in VDR, mediated gene expression. Under nonproliferating conditions, AR would therefore compete with VDR for 2 WINAC components and 4 transcriptional activators. *SRC1 is a general transcription activator for steroid receptors and also component of this pathway. SRC1 was not identified by mass spectrometry, but AR-associated in Western blots of FLAG-purifications from nuclear extracts of proliferating and nonproliferating P17 cells (Figures 4 and 3(b)).

Common pathways enriched for or overrepresented in proliferating and nonproliferating AR FLAG purifications were the biological processes “transcriptional regulation” and “gene expression”. Considering that the androgen receptor is classified as a transcription factor, high scores in those categories are expected. Scoring surprisingly high were BIOCARTA pathways not involving AR at all such as “chromatin remodelling by hSWI/SNF ATP-dependent complexes” and “control of gene expression by vitamin D receptor (VDR)” (Figure 5), which involves the WINAC chromatin-remodelling complex. Both WINAC and SWI/SNF complexes have BAF components (Brg1-associated factors). The requirement of the BAF complexes has been shown *in vitro* for ligand-dependent transactivation by nuclear hormone receptors, such as vitamin D3 receptor, retinoid X receptor, and peroxisome proliferator-activated receptor PPAR-*γ* [16]. It has also been shown *in vivo* for reconstitution of glucocorticoid-receptor-(NR3C1-) dependent transcription [17], chromatin remodelling on interferon and virus inducible genes [18] and in neural development with a subunit switch in the npBAF (neural progenitors-specific) chromatin remodelling complex, essential for the transition from neural stem/progenitors to postmitotic neurons [19] but has never been associated with AR. Also the Vitamin D3 receptor has not been identified as AR-interacting protein neither in our purification (data not shown) nor by others. It is however possible that AR transactivation might be stimulated by components of the WINAC complex. The VDR and the AR could therefore compete for shared coregulators, which would explain the observation that AR stimulation by androgens suppresses VDR [20, 21], while AR downregulation by siRNA stimulates VDR levels in LnCAP cells [21]. WINAC complex components might be potential coplayers in AR transactivation, which could be tested with siRNA cotransfection experiments in our cell line. G-profiler, which does not offer a tool such as the BIOCARTA, also identified BAF components as being overrepresented. Another overrepresented pathway is the prostate cancer pathway, which is picked up by both bioinformatics tools. G-profiler does not identify EP300 as a gene involved in prostate cancer as it is done by DAVID (Table 3 bottom). Also the “Kegg cancer pathway” is picked up by both bioinformatics tools: g-profiler identifies the same list of genes among the known AR-interacting proteins as “DAVID”, only that RB1 is not included in the g-profiler gene list (Table 3 bottom).

For male genital development and PAIS relevant biological processes are “embryo development”, which is here represented with 13 genes identified by g-profiler and 11 identified by DAVID. Of those 10 are overlapping (Table 4). The embryonic genes identified by DAVID are restricted to chordate development and do not include CTNNB1, HDAC1 and TGFBI1, although CTNNB1 and HDAC1 “knock outs” in mouse have been shown to result in developmental phenotypes [22, 23]. G-profiler did not pick up EP300. A role of EP300 in patterning and development was suggested by studies in mice in which EP300 expression was disrupted [24]. Both bioinformatic tools David and g-profiler complemented each other in this study in identifying androgen receptor regulated pathways and biological processes.

In future we aim to replace the endogenous AR with N-TAP-mAR. We will hopefully be able to apply the FLAG purification protocol tested in this study to identify differences in the proteome caused by the respective AR mutation. The protein purification approach taken here and shown to identify differences in co-factor recruitment of AR under proliferating and nonproliferating conditions is encouraging to undertake further experiments aiming to identify specific interaction protein profiles for AR mutants associated with PAIS.

## Figures and Tables

**Table:** (a)

33 only	coA/coR	Score: P17/*PC1 *(slice)	Uniprot	37 only	coA/coR	Score: P17 (slice)	Uniprot
KDM3A	CoA	52 (3)	Q6PCM1	KDM5B	coA	93 (5)	Q80Y84
WHSC1	CoA	252 (5)	Q8BVE8	DAPK3	coA	67 (1)	O54784
MED17	CoA	66 (2)	Q8VCD5	PIAS1	coA/CoR	38 (2)	O88907
MED24	CoA	156 (3)	A6PW47	MED1	coA	44 (6)	Q925J9
ARID1B	CoA	38 (6)	E9Q4N7	GATA3	coA	55 (1)	P23772
SRC	—	57 (1)	Q2M4I4	SP1	coA	81 (3)	O89090
*SMARCC1*	*CoA*	*1112/60 *(5)	Q3UNN4	TGFBI1	coA	32 (1)	Q62219
SMARCD1	CoA	39 (1)	Q61466	TRIM24	coA	165 (4)	Q64127
NPM1	CoA	438 (6)	Q5SQB0	FKBP5	coA	29 (1)	Q64378
GTF2F1	—	89 (2)	Q3THK3	RANBP10	coA	53 (2)	Q6VN19
				CALCOCO1	coA	156 (3)	Q8CGU1
				PSPC1	coA	35 (2)	Q8R326
				GAK	coA	397 (5)	Q99KY4
				NCOA2	coA	68 (5)	Q61026
				NR3C1	coR	97 (3)	Q06VW2
				AP-1	coA/CoR	56 (2)	Q3TXG4
				RB1	coA	55 (5)	Q3URY9
				NF1	CoA	46 (7)	Q04690-1

**Table:** (b)

Common	coA/coR	Score 33°C: P17*/PC1* (slice)	Uniprot	Score 37°C: P17 (slice)
STAT3	CoA	41 (3)	P42227	90 (3)
DAXX	CoR	66 (4)	Q3UIV3	70 (4)
ARID1A	CoA	107 (5)	A2BH40	78 (5)
*HDAC1 *	*CoR*	* 428/52 *(2)	*O09106*	251 (2)
BRD7	CoR	169 (3)	O88665	50 (3)
*HSP90*	*CoR*	* 719/250 *(3)	*P07901*	1089 (3)
GSN isoform1 [15]	CoA ?	542 (4)	P13020-1	516 (4)
*GSN isoform2 *[15]	*CoA ?*	*1879/624 *(3)	*P13020-2*	1688 (3)
CALR	CoR	148 (1)	P14211	612 (1)
HSPA1B	CoA	434 (2)	P17879	582 (2)
AR	CoA	115 (4)	P19091	200 (4)
*XRCC6*	*CoA*	*1208/85 *(2)	*P23475*	1380 (2)
MCM3	CoA	728 (3)	P25206	1476 (3)
*XRCC5*	*CoA*	*1727/49 *(3)	*P27641*	1717 (3)
*PA2G4*	*CoR*	* 89/257 *(1)	*P50580*	54 (1)
*ACTN4*	*CoA/CoR*	*3147/3229 *(3)	*P57780*	3112 (3)
ACTB	CoA	1386 (1)	P60710	1106 (1)
DNAJA1	CoR	173 (1)	P63037	371 (1)
PRKDC	CoA	1134 (7)	P97313	739 (7)
*CTNNB1*	*CoA*	* 242/44 *(3)	*Q02248*	242 (3)
*SMARCA4 *	*CoA*	*1841/73 *(6)	*Q3TKT4*	1449 (6)
MYST2	CoR	437 (2)	Q5SVQ0	36 (2)
*KHDRBS1*	*CoR*	* 137/100 (*2)	*Q60749*	274 (2)
*DDX5*	*CoA*	*1099/753 *(2)	*Q61656*	1150 (2)
SMARCA2	CoA	727 (6)	Q6DIC0	789 (6)
PPP2R1A	CoR	433 (1)	Q76MZ3	97 (1)
*RBM14*	*—*	* 232/201 *(2)	*Q8C2Q3*	373 (2)
COBRA1	CoR	50 (6)	Q8C4Y3	40 (6)
ATAD2	CoA	405 (5)	Q8CDM1	2221 (5)
KIAA1967	CoA	39 (4)	Q8VDP4	92 (4)
*SFPQ*	*CoA/CoR*	* 766/446 *(3)	*Q8VIJ6*	490 (3)
PRPF6	CoA	259 (3)	Q91YR7	218 (3)
*NONO*	*CoA/CoR*	* 625/197 (*1)	*Q99K48*	472 (1)
PELP1	CoA	261 (5)	Q9DBD5	504 (5)
SART3	CoR	286 (4)	Q9JLI8	863 (4)
NCOR2	CoR	107 (7)	Q9WU42	95 (7)
PSMC3	CoA	148 (1)	A2AGN7	247 (1)
EHMT2	CoA	66 (5)	A2CG76	282 (5)
KDM1A	CoA	210 (4)	A3KG93	100 (4)
ZFP318	CoR	35 (3)	B0V2M3	30 (3)
EP300	CoA	31 (7)	B2RWS6	107 (7)
*FLNA*	*CoA/CoR*	*6598/1307 *(7)	*B7FAU9*	6972 (7)
BRD8	CoA	62 (5)	Q8R3B7	164 (5)
*SUPERVILLIN*	*CoA*	* 53/44 *(6)	*Q8K4L3*	439 (6)
*DDX17*	*—*	* 584/336 *(2)	*Q3U741*	645 (2)
HDAC6	CoA	99 (4)	Q3UG37	142 (4)
*TRP53*	*CoA*	*1026/71 *(1)	*Q80ZA1*	1090 (1)
SRCAP	CoA	57 (7)	Q8BKT0	82 (7)

**Table 2:** Gene lists representing the biological processes (BP) “regulation of transcription” and “gene expression” identified by the bioinformatics tools “DAVID” and “g-profiler” as being overrepresented among the known AR-interacting proteins of the N-TAP-mAR purification. Listed are the interacting proteins, the AR is in bold face, that were unique for the proliferating cells (33), interacting proteins that were unique for the nonproliferating cells (37), and interacting proteins common to both (common).

Regulation of transcription (BP) DAVID			Gene expression (BP) g-profiler		
33	Common	37	33	Common	37
KDM3A	EP300			EP300	
	SMARCA4			SMARCA4	
	**AR**			**AR**	
WHSC1		MED1	WHSC1		MED1
	CTNNB1			CTNNB1	
MED17		NR3C1	MED17		NR3C1
	PRPF6			PRPF6	
	KDM1A			KDM1A	
MED24		SP1	MED24		SP1
	TRP53			TRP53	
	MYST2			MYST2	
	DDX5		SRC	DDX5	
	DAXX			DAXX	
SMARCC1		GATA3	SMARCC1		GATA3
	RBM14		SMARCD1	RBM14	
	BRD7		NPM1	BRD7	
GTF2F1		CALCOCO1	GTF2F1		CALCOCO1
	EHMT2			EHMT2	
	FLNA			FLNA	
		KDM5B			KDM5B
	NONO			NONO	
	NCOR2			NCOR2	
		NCOA2			NCOA2
	PA2G4			PA2G4	
		RB1			RB1
	STAT3			STAT3	
	HDAC1			HDAC1	
	SFPQ			SFPQ	
		TRIM24			TRIM24
	HDAC6			PRKDC	
	COBRA1			CALR	
	ATAD2			XRCC6	
	MCM3			SART3	
	BRD8				
	SMARCA2				
	KHDRBS1				
	ZFP318				
		TGFB1l			
		PSPC1			
		PIAS1			

**Table 3:** Top half: gene lists representing the BIOCARTA pathways “telomeres, telomerase, cellular aging, and immortality”, “chromatin remodeling by hSWI/SNF ATP-dependent complexes” and “control of gene expression by vitamin D receptor” found to be overrepresented among the known AR-interacting proteins identified in the N-TAP purifications by the bioinformatics tool DAVID. Bottom half: gene lists representing the “Kegg pathway”  “pathways in cancer”, and “prostate cancer” identified as being overrepresented among the known AR-interacting proteins identified in the N-TAP purifications by the bioinformatics tool “DAVID” and “g-profiler”. Listed are the interacting proteins that were unique for the proliferating cells (33), interacting proteins that were unique for the nonproliferating cells (37), and interacting proteins common to both (common). The AR is bolded.

Cellular aging, telomeres,			Chromatin remodelling by SWI/SNF			Control of gene expression by					
immortality (DAVID)			ATP-dependent complexes (DAVID)			vitamin D receptor (DAVID)					
33	Common	37	33	Common	37	33	Common	37			
							EP300				
					NR3C1			MED1			
				ARID1A			ARID1A				
	HSP90		ARID1B								
			SMARCD1			SMARCD1					
		RB1	SMARCC1			SMARCC1					
	XRCC5			SMARCA4			SMARCA4				
	XRCC6			ACTB				NCOA2			
	TRP53				NF1						

pathways in cancer			pathways in cancer			prostate cancer			prostate cancer		
DAVID			(g-profiler)			(DAVID)			(g-profiler)		
33	common	37	33	common	37	33	common	37	33	common	37

	EP300			EP300			EP300				
	**AR**			**AR**			**AR**			**AR**	
	CTNNB1			CTNNB1			CTNNB1			CTNNB1	
		DAPK3			DAPK3						
	HSP90			HSP90			HSP90			HSP90	
		PIAS1			PIAS1						
		RB1						RB1			
	STAT3			STAT3							
	HDAC1			HDAC1							
	TRP53			TRP53			TRP53			TRP53	

**Table 4:** Gene lists for the biological processes (BP) “chordate embryonic development” and “embryo development” found to be overrepresented among the known AR-interacting proteins identified in the N-TAP purifications from the 37°C samples but not the 33°C samples by the bioinformatics tool “DAVID” and “g-profiler”. Listed are the interacting proteins that were unique for the nonproliferating cells (37) and interacting proteins common to both (common). The AR is bolded. AR-interacting proteins identified only in the 33°C samples are not involved in biological processes addressed here. No counterpart for the biological process “anatomical structure morphogenesis” could be identified by DAVID.

Chordate embryonic			Embryo development (BP)			Anatomical structure morphogenesis (BP)		
Development (BP) DAVID			g-profiler			g-profiler		
33	Common	37	33	Common	37	33	Common	37
	NCOR2			NCOR2			NCOR2	
	SMARCA4			SMARCA4			SMARCA4	
	**AR**			**AR**			**AR**	
		MED1			MED1			MED1
		NF1			NF1			NF1
		SP1			SP1			SP1
	PSMC3			PSMC3				
	PRKDC			PRKDC			PRKDC	
	KDM1A			KDM1A				
	TRP53			TRP53			TRP53	
				CTNNB1			CTNNB1	
					TGFBI1			TGFBI1
								NR3C1
				HDAC1			HDAC1	
							GSN	
							GATA3	
	EP300						ACTB	
							STAT3	
